# Cholecalciferol Injections Are Effective in Hypovitaminosis D After Duodenal Switch: a Randomized Controlled Study

**DOI:** 10.1007/s11695-018-3307-8

**Published:** 2018-06-04

**Authors:** Hella Hultin, Katharina Stevens, Magnus Sundbom

**Affiliations:** Department of Surgical Sciences, Uppsala University, Entrance 70, SE-751 85 Uppsala, Sweden

**Keywords:** Hypovitaminosis D, Cholecalciferol, Biliopancreatic diversion, Duodenal switch

## Abstract

**Background:**

By treating obesity, one of the major epidemics of this past century, through bariatric surgery, we may cause complications due to malnourishment in a growing population. At present, vitamin D deficiency is of interest, especially in patients with inferior absorption of fat-soluble nutrients after biliopancreatic diversion with duodenal switch (BPD/DS).

**Methods:**

Twenty BPD/DS patients, approximately 4 years postoperatively, were randomized to either intramuscular supplementation of vitamin D with a single dose of 600,000 IU cholecalciferol, or a control group. Patients were instructed to limit their supplementation to 1400 IU of vitamin D and to avoid the influence of UV-B radiation; the study was conducted when sunlight is limited (December to May).

**Results:**

Despite oral supplementation, a pronounced deficiency in vitamin D was seen (injection 19.3; control 23.2 nmol/l) in both groups. The cholecalciferol injection resulted in elevated 25[OH]D levels at 1 month (65.4 nmol/l), which was maintained at 6 months (67.4 nmol/l). This resulted in normalization of intact parathyroid hormone (PTH) levels. No changes in vitamin D or PTH occurred in the control group.

**Conclusions:**

In BPD/DS patients, having hypovitaminosis D despite full oral supplementation, a single injection of 600,000 IU of cholecalciferol was effective in elevating vitamin D levels and normalizing levels of intact PTH. The treatment is simple and highly effective and thus recommended, especially in cases of reduced UV-B radiation.

## Background

The global trends of obesity predict that the number of patients treated with bariatric surgery will continue to grow, and possibly begin to include patients as young as teenagers [1, 2]. Of the bariatric procedures performed in Sweden and elsewhere, biliopancreatic diversion with duodenal switch (BPD/DS) results in the most significant weight loss, and is therefore often reserved for patients with severe morbid obesity (BMI > 50 kg/m^2^). BPD/DS obtains weight loss through its dual mechanisms of restriction and malabsorption. The restrictive mechanism arises from the gastric sleeve, and the malabsorptive mechanism by leaving only 100 cm of the distal ileum for absorption of fat-soluble nutrients (the so-called common limb). However, in addition to massive weight loss, the procedure is associated with a significantly higher prevalence of nutrient deficiencies, including but not limited to vitamin D [3, 4]. Despite recommended multivitamin supplementation, including 2000 IU vitamin D3 daily, two thirds of BPD/DS patients were found to have vitamin D deficiency [5].

Normal physiological functioning is highly dependent upon calcium, which relies upon a complex feedback system that regulates the levels in blood and extracellular fluids. The parathyroid gland releases parathyroid hormone (PTH) in response to a decrease in serum calcium concentration which in turn signals for increased uptake of calcium in the intestine. This uptake is strictly dependent upon an adequate level of vitamin D [6]. Vitamin D is a fat-soluble prohormone, present in various forms and not active until hydroxylation by 1-alpha-hydroxylase. The inactive storage form of vitamin D is 25-hydroxycholecalciferol (25[OH]D), which is widely accepted when measuring levels of vitamin D in the clinical setting [7]. A deficiency in vitamin D is a causative agent of rickets, secondary hyperparathyroidism, and osteoporosis. Other health concerns associated with vitamin D deficiency include neuropsychiatric disorders, cardiovascular disease, diabetes, and cancer.

Hypovitaminosis D is often described in the obese population, where plausible causes include limited sun exposure, poor nutrition, and sequestration of vitamin D in adipose tissue [7–9]. Massive weight loss through bariatric surgery has been shown to restore levels initially; however, post-operative hypovitaminosis D is equally commonly described in the long term, despite various types of oral supplementation [10–13]. A previous study conducted by our group demonstrated that gastric bypass patients could be effectively treated for vitamin D deficiency with UV-B treatment three times a week [14]. However, as the treatment was considered tiresome, compliance as well as vitamin D levels decreased during the course. As BPD/DS patients often have more severe vitamin D deficiencies, and the altered anatomy undermines oral vitamin D absorption [15], alternative treatments, especially in patients with deficiencies in spite of full oral supplementation, is warranted.

The aim of this study was to evaluate intramuscular administration of cholecalciferol in patients with hypovitaminosis D after BPD/DS, despite standardized oral supplementation with 1400 IU cholecalciferol.

## Materials and Method

### Patients

Seventy-three patients having undergone BPD/DS at Uppsala University Hospital between 2008 and 2010 were invited to participate in the study by mail. By the order of acceptance, participants were randomly assigned 1:1 to either intramuscular supplementation of vitamin D or to a control group. Both groups left blood samples at baseline, and at 1, 3, and 6 months.

Participants were excluded from the study if they had recently or planned upcoming travels to sunny climates or were concomitantly using potentially interacting drugs, such as thiazides, corticosteroids, phenytoin, cholestyramine, phenobarbital, and/or cardiac glycosides. Patients treated for hypercalcemia, osteoporosis, primary hyperparathyroidism, and/or renal failure were also excluded as well as pregnant and breastfeeding patients.

None of the patients had undergone bariatric surgery prior to their BPD/DS procedure. Our BPD/DS procedure entails a linear stapled gastric sleeve resection and the creation of a 250-cm alimentary limb, of which the last 100 cm constitutes the common limb (distal ileum). The duodeno-ileal anastomosis is hand sewn end-to-side, using the total width of the duodenal bulb [16].

As demonstrated in Table 1, there were no differences between the injection group and controls concerning gender, age, pre- and post-operative BMI, or time after surgery. Major comorbidities, including sleep apnea (*n* = 1), diabetes mellitus (*n* = 0), hypertension (*n* = 0), dyslipidemia (*n* = 0), and depression (*n* = 2), were rare at the time of the study.

**Table 1:** Demographics of the patient population at time of entry into the study, i.e., baseline. Data is presented as a percentage of the total and median values (interquartile range)

	Injection group (*n* = 11)	Control group (*n* = 9)	*p* value
% female	61.5%	72.7%	0.88
Age (years)	42.0 (7.0)	38.0 (8.0)	0.71
Years postop	4.2 (1.7)	4.9 (1.8)	0.15
Preop BMI (kg/m^2^)	54.5 (4.6)	54.9 (2.4)	0.60
BMI at study (kg/m^2^)	33.1 (5.2)	35.0 (8.6)	0.66
Albumin (g/L)	36.0 (5.5)	37.0 (3.0)	0.88
Creatinine (μmol/L)	58.0 (13.5)	56.0 (12.0)	0.82

The *p* value is calculated using the Mann-Whitney non-parametric *U* test

Vitamin D levels are subjected to considerable seasonal variation, where in northern Europe the lowest levels in a geographically stationary population measure in April and the highest in October [15]. This study was conducted from December to May when sunlight is limited in Sweden to avoid the influence of UV-B radiation, as it is a major source in the process of vitamin D synthesis. The injection group received a single dose of 600,000 IU cholecalciferol vitamin D3 Streuli® (Streuli Pharma AG, Uznach); the dosage was chosen according to earlier studies [14, 17]. Patients in both groups were instructed to limit their total diet supplementation to 1400 IU of vitamin D. Diet was not otherwise regulated in either group.

### Laboratory Tests

Both groups left blood samples at the start and at 1, 3, and 6 months. Blood samples were analyzed for 25[OH]D, intact PTH, and serum calcium. Albumin and creatinine levels were analyzed at baseline only. The analysis of 25[OH]D was performed at Vitas Labs, Oslo, using a HPLC method (Agilent Technologies, Palo Alto, CA, USA). The remaining analyses were performed by the clinical chemistry laboratory at the University Hospital in Uppsala. Intact plasma PTH (normal range 1.1–6.9 pmol/L) was measured with a chemiluminescent solid-phase two-site immunoassay using an IMMULITE 2500 (Diagnostics Product Corporation, Los Angeles, USA). Serum calcium was measured spectrophotometrically with a complexometric method using orthocresolphtalein (normal range 2.15–2.50 mmol/L). Serum albumin was determined by spectrophotometry using Bromine Bresol Breen (normal range 37–48 g/L). Serum creatinine was measured by spectrophotometry using Jaffe’s reaction (normal range 60–106 μmol/L).

### Statistics

Unless stated, values are presented as median and interquartile range (IQR). Significance was established using the Mann-Whitney non-parametric *U* test. A *p* value < 0.05 was considered significant. A sample size calculation was based on the increase to 80 mmol/L (SD 18) in 25[OH]D, this in response to an identical dose of intramuscular cholecalciferol as used in the study by Einarsdóttir [17] and the assumption that the present BPD/DS patients had a baseline value no greater than 50 (as found in our previous work in gastric bypass patients [14]. In order to demonstrate an increase from 50 to 80 with an SD of 18 and a power index of 95% at *p* < 0.05, the required sample size was nine patients in each group. The study was approved by the local ethics committee at the University of Uppsala (Reference 2012/201) and the Medical Products Agency, responsible for surveillance of the development, manufacturing, and marketing of drugs nationally (EudraCT 2012-002217-19).

## Results

Despite oral supplementation post-surgery, both groups had 25[OH]D levels below 50 nmol/l (injection 19.3; control 23.2 nmol/l) after more than 4 years postoperatively. As demonstrated in Table 2 the cholecalciferol injection resulted in elevated 25[OH]D levels at 1 month (65.4 vs. 29.2 nmol/l in controls, *p* < 0.01) and maintained at that level until the conclusion of the study, 6 months post-administration (67.4 vs. 29.2 nmol/l, *p* = 0.04).

**Table 2:** Effect of a single cholecalciferol injection on 25[OH] vitamin D, PTH, and calcium from baseline to 6 months

	Baseline (*n* = 11 and 9, resp.)	1 month (*n* = 11/8)	3 months (*n* = 7/6)	6 months (*n* = 9/5)
25[OH]D (nmol/l)				
Injection group	19.3(9.3)	65.4 (6.3)	66.6 (11.0)	67.4 (16.1)
Controls	23.2 (22.5)	29.2 (13.4)	24.0 (23.0)	29.2 (31.4)
*p* value	0.30	< 0.01	0.03	0.04
Intact PTH (pmol/l)				
Injection group	12.3 (9.9)	6.4 (2.1)	7.3 (4.0)	9.6 (6.2)
Controls	9.5 (6.8)	8.5 (4.3)	8.4 (2.4)	9.1 (1.8)
*p* value	0.20	0.15	0.23	0.90
Calcium (mmol/l)				
Injection group	2.2(0.14)	2.18 (0.13)	2.22 (0.13)	2.19 (0.06)
Controls	2.2 (0.10)	2.20 (0.20)	2.20 (0.20)	2.20 (0.10)
*p* value	0.33	0.40	0.84	1.0

Data is presented as median value (interquartile range) and was compared to the Mann-Whitney non-parametric *U* test

**Fig. 1:**
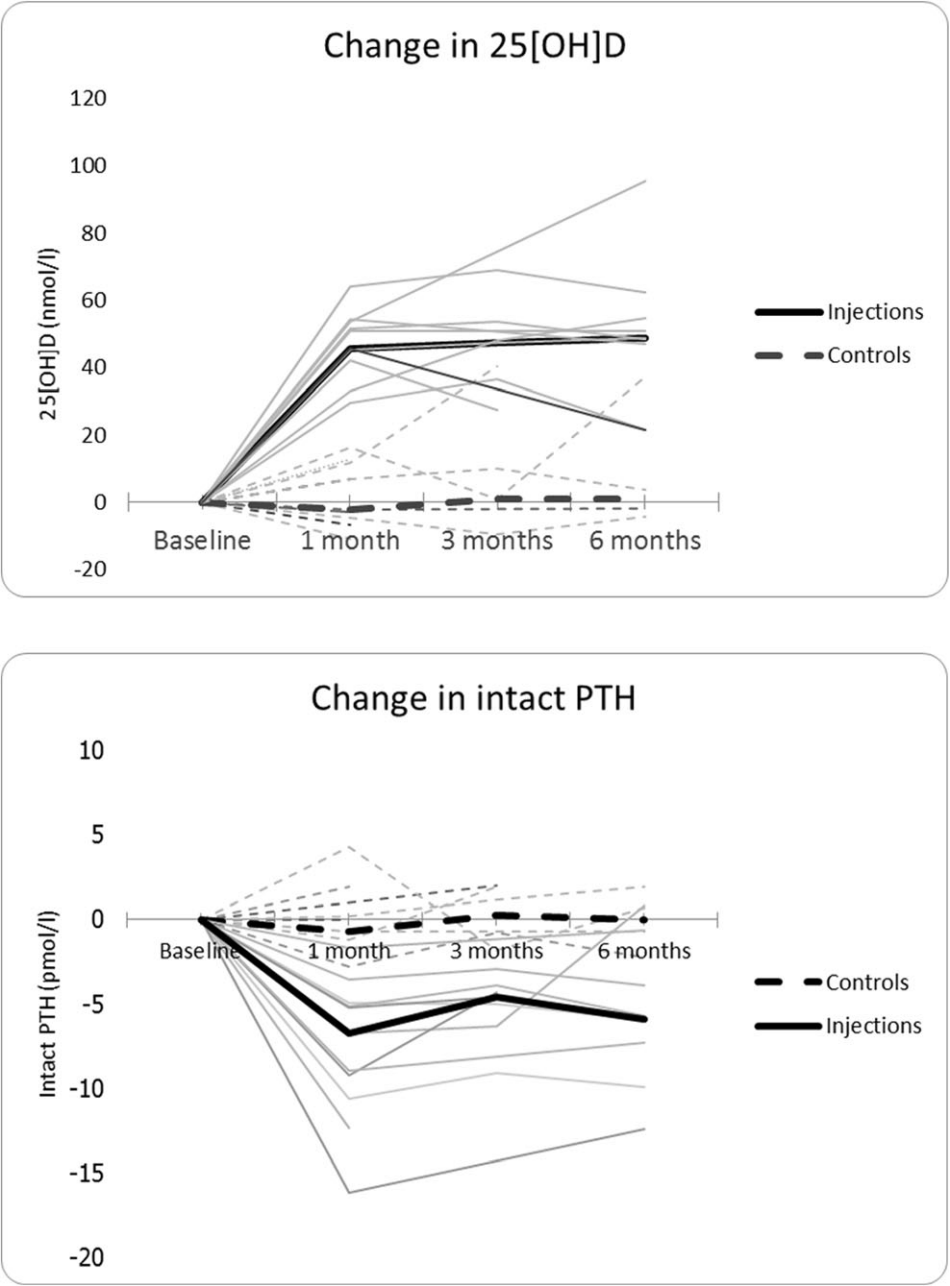
The changes in 25[OH]D and PTH levels for all patients in the two groups

Both groups maintained a normal serum calcium level; however, the elevated PTH levels suggest that compensation through PTH-mediated mechanisms had been necessary. All patients in the treatment group obtained normalized PTH levels up to 6 months post-injection. The changes in 25[OH]D and PTH levels for all patients in the two groups are demonstrated in Fig. 1.

No complications, e.g., hypercalcemia with severe thirst and polyuria, or impaired renal function due to vitamin D intoxication occurred.

## Discussion

A single injection of 600,000 IU of cholecalciferol was effective in restoring and maintaining normal levels of vitamin D and PTH for 6 months in BPD/DS patients with hypovitaminosis D, despite full oral supplementation. No complications occurred during the study period. This simple and effective treatment is therefore a recommended substitute to oral vitamin D supplements, especially in patients receiving limited UV radiation.

### Vitamin D

Calcium homeostasis is obtained through the interplay between multiple factors and organs. To maintain the narrow range of acceptable serum concentration, the body relies upon the skeleton as the ultimate calcium storage source to compensate for deficient amounts obtained via the skin and the alimentary tract. The parathyroid glands release PTH in response to low serum calcium concentrations which in turn signals for increased vitamin D activation in the kidney, increased uptake of vitamin D in the intestine, and metabolism of bone to release stored calcium. Vitamin D promotes the uptake of calcium from the diet at the duodenal mucosa by increasing the number of calcium channels. Vitamin D is therefore essential to maintain calcium homeostasis.

The definition of vitamin D sufficiency remains a topic of discussion; however, commonly used cutoffs are 75 nmol/l for insufficiency and 25 nmol/l for deficiency [18]. Levels of 25[OH]D below 75 nmol/l is associated with muscle weakness, increased risk of fall, and type 2 diabetes mellitus, and levels below 25 nmol/l is further associated with diabetes mellitus type 1, cardiovascular disease, neoplasms, fibromyalgia, chronic fatigue, neuropsychiatric disorders, and secondary hyperparathyroidism followed by osteoporosis [19–21].

### Bone Health

Few retrospective studies have been published concerning incidence of fractures and prevalence of osteoporosis among bariatric patients; however, of these few, a study conducted in Quebec, Canada, of 12,676 postoperative bariatric patients versus obese and non-obese patients showed that bariatric patients have a significantly increased relative risk of fracture. The biliopancreatic diversion procedure was associated with the most significant relative risk of fracture when compared to other bariatric procedures [22]. A study conducted in Minnesota analyzed retrospectively 258 post-bariatric surgery patients for incidence of fracture and found a twofold increased risk of fracture of the hip/spine/wrist, where most fractures occurred at least 5 years postoperatively [23].

### BPD/DS

The BPD/DS surgery is an effective weight loss procedure because of its two working mechanisms: reduced intake and reduced absorption of ingested nutrients. The uptake of fat-soluble substances can only occur in the most distal 100 cm of the distal ileum (common limb), after mixing with bile, making BPD/DS the most malabsorptive bariatric procedure [24]. Moreover, the bypass of the duodenum, which has the highest density of vitamin D receptors, results in reduced effect of oral vitamin D supplements [25]. A study of 43 BPD/DS patients demonstrated deficiencies of micronutrients 5 years postoperatively where the most significant deficiency was vitamin D (76.7%) despite recommended multivitamin supplementation including 2000 IU vitamin D3 daily [5]. At the time of the study, the recommended vitamin supplementation for patients having undergone BPD/DS at Uppsala University Hospital included 1400 IU cholecalciferol daily. Awaiting national consensus regarding supplementation post bariatric surgery, dosage recommendations have varied between centers. Thus, patients having had BPD/DS require life/long monitoring of micronutrients at a specialized bariatric center and possibly better micronutrient supplementation.

### Limitations

The strengths of this study include the same geographic location and thereby controlled UV-B radiation, all Caucasian participants, identical length of the common limb (100 cm) in all patients, and a modern mode of vitamin D laboratory analysis. Moreover, patients were not allowed to travel to sunny climates or use medications known to alter calcium homeostasis, such as vitamin D analogues, cholestyramine, phenobarbital, phenytoin, corticosteroids, thiazides, or heart glycosides. Although significant statistical differences were found, the study groups were small. The fact that 25[OH]D levels remained unchanged in the controls support the notion that per oral absorption of vitamin D is reduced in BPD/DS. Due to the fact that the participants in the study were only of Caucasian ethnicity, our results might not be relevant to patient groups of other ethnicities.

## Conclusions

In BPD/DS patients having hypovitaminosis D despite full oral supplementation, a single injection of 600,000 IU of cholecalciferol was effective in normalizing intact PTH and vitamin D levels. The treatment is simple and highly effective and thus recommended, especially in populations subjected to reduced UV-B radiation.
